# Expression of Mcm2, geminin and Ki67 in normal oral mucosa, oral epithelial dysplasias and their corresponding squamous-cell carcinomas

**DOI:** 10.1038/sj.bjc.6604967

**Published:** 2009-03-17

**Authors:** A Torres-Rendon, S Roy, G T Craig, P M Speight

**Affiliations:** 1 Department of Oral and Maxillofacial Pathology, School of Clinical Dentistry, University of Sheffield, Claremont Crescent, Sheffield S10 2TA, UK

**Keywords:** oral cancer, dysplasia, Mcm2 protein, geminin protein, cell-cycle proteins

## Abstract

Proteins necessary for the normal regulation of the cell cycle include minichromosome maintenance protein 2 (Mcm2) and geminin. These are overexpressed in several premalignant and malignant tumours. The Mcm2/Ki67 ratio can be used to estimate the population of cells that are in early G_1_ (licensed to proliferate), and the geminin/Ki67 ratio can determine the relative length of G_1_. A high ratio indicates a short G_1_ and a high rate of cell proliferation. Mcm2 and geminin have been scarcely explored in oral epithelial dysplasia (OED) and oral squamous-cell carcinoma (OSCC). The purpose of this study was to identify the expression pattern of Mcm2, Ki67 and geminin in normal oral mucosa (NOM), OED and their subsequent OSCC, to determine if expression could help predict the prognosis of OED. Paraffin sections of 41 OED cases that progressed to carcinoma, 40 OED without malignant progression, 38 OSCC and 15 NOM were immunostained with antibodies against Mcm2, geminin and Ki67. Labelling indices (LIs) increased progressively from NOM, OED and OSCC (Mcm2, *P*<0.001; geminin, *P*<0.001 and Ki67, *P*<0.001). In all the OED cases (*n*=81) the levels of expression of Mcm2 (LI, 73.6), geminin (LI, 24.4) and Ki67 (LI, 44.5) were elevated indicating a constant cell-cycle re-entry. When the OED groups were compared, Mcm2 protein expression was higher in the OED with malignant progression (*P*=0.04), likewise there was a significant increase in the Mcm2/Ki67 and geminin/Ki67 ratios (*P*=0.04 and 0.02 respectively). Mcm2 and geminin proteins seem to be novel biomarkers of growth and may be useful prognostic tools for OED.

Oral squamous-cell carcinoma (OSCC) represents the majority of malignant lesions of the oral cavity. Despite refinement of surgical techniques in the past few decades once invasive cancer is formed, the prognosis is poor with an average 5-year survival rate of 40% of affected patients (Mork, 1998; Diaz *et al*, 2003). Early diagnosis of OSCC can improve patient survival and reduce significantly the high mortality rates. It is thought that most, if not all, OSCC are preceded by a period during which the affected epithelium shows evidence of epithelial dysplasia, although this may not always be clinically apparent (Napier and Speight, 2008). Currently, there are no markers that can reliably predict malignant progression in oral dysplastic lesions.

Cell-cycle regulatory proteins, including those that promote initiation of DNA synthesis, such as minichromosome maintenance proteins (MCM) and those that help to regulate cell differentiation and cell proliferation such as geminin are deregulated in a number of malignant lesions from different human organs. These have been shown to be effective markers for the early detection of cancer, particularly in samples from suspected epithelial malignancies (Freeman *et al*, 1999; Stoeber *et al*, 1999; Gonzalez *et al*, 2004).

Minichromosome maintenance proteins (Mcm 2–7) are a pre-requisite for DNA replication and cell-cycle initiation (Kearsey and Labib, 1998) and are expressed throughout the whole cell cycle including cells leaving G_0_ to enter into the early G_1_ phase. This characteristic is not found in Ki67, which is widely used to assess proliferation; and therefore MCM proteins specifically Mcm5 and Mcm2 have been proposed as potential prognostic markers in dysplastic lesions and cancer (Freeman *et al*, 1999; Stoeber *et al*, 1999; Alison *et al*, 2002; Going *et al*, 2002).

Geminin, a novel proliferation marker is present from the G_1_–S transition to early M phases (McGarry and Kirschner, 1998) and helps to regulate cell-cycle initiation by impeding a second pre-replication complex assembly once replication has started (McGarry and Kirschner, 1998; Tachibana *et al*, 2005). It has also been suggested that geminin levels may be altered in cancer cells as it has been found to be overexpressed in several cancer cell lines (Xouri *et al*, 2004). In addition, elevated expression has been linked with a poor clinical outcome in renal cell carcinomas (Dudderidge *et al*, 2005), and has been shown in a range of other malignancies (Wohlschlegel *et al*, 2002; Shetty *et al*, 2005).

Ki67 is present throughout the complete cell cycle with the exception of early G_1_ phase (Gerdes *et al*, 1984). When Mcm2 and geminin are evaluated along with Ki67, the combination may provide extra information about proliferation rates in epithelial compartments (Quaglia *et al*, 2006). The geminin/Ki67 ratio can potentially evaluate the relative length of G_1_. The higher the ratio, the shorter G_1_ indicating an increased proliferation rate and therefore a poor prognosis, as shown in oligodendroglial tumours (Wharton *et al*, 2004). Another suggested prognostic parameter is the Mcm2/Ki67 ratio (Shetty *et al*, 2005), which can estimate the proportion of cells that are licensed to proliferate (early G_1_).

The purpose of this study was to determine the immunohistochemical expression pattern of these proteins in normal oral mucosa (NOM), oral epithelial dysplasia (OED) and OSCC, and to determine if abnormal expression could serve as a prognostic marker of malignant progression in OED.

## Material and methods

The study design was a retrospective case–control study identifying lesions of OED that had progressed to malignancy and comparing the protein expression with a cohort of closely matched lesions that had not progressed at the time of sampling. Ethical approval was obtained from the South Sheffield Research Ethics Committee (SSREC/03/206).

### Patient selection

From a total of over 1000 cases of OED in the archives of the diagnostic service of the Oral and Maxillofacial Pathology Department of the University of Sheffield, 41 were identified that had progressed to OSCC. For each case the first biopsy showing evidence of dysplasia was chosen for analysis. From the remaining cases 40 closely matched OED that had not progressed to carcinoma after a minimum follow-up of 4.5 years were selected. Of the 41 cases that had progressed, 38 of the OSCC were available for analysis.

The final total of 134 cases selected for analysis comprised 41 OED that had progressed to carcinoma, 40 OED that did not show malignant progression, 38 OSCC from the 41 cases that had progressed and 15 samples of NOM. If multiple biopsies for OED were present from the same patient, the first OED biopsy was selected; if the first biopsy was from a different site to that of the subsequent OSCC, then, if available, the next biopsy that matched the same site as the subsequent OSCC was chosen. Synchronous lesions were excluded by only selecting cases where the minimum time between the diagnosis of OED and carcinoma was 6 months. OED and OSCC were histopathologically graded by two experienced oral pathologists (PMS and GTC) according to the latest WHO guidelines (Barnes *et al*, 2005).

### Immunohistochemistry

The antibodies used in this study were anti-Mcm2 (BM28) mouse anti-human monoclonal antibody (clone 46; BD Transduction Laboratories, Lexington, KY, USA), geminin 95 (Gem95) polyclonal rabbit anti-human (Wharton *et al*, 2004) antibody and anti-human Ki67 monoclonal mouse antibody (clone MIB-1) (Dako, Glostrup, Denmark). All these proteins have been previously characterised (Gerdes *et al*, 1984; Todorov *et al*, 1994; Lea *et al*, 2003; Wharton *et al*, 2004).

Paraffin-embedded formalin-fixed tissue sections (4 *μ*m) were de-waxed in xylene and re-hydrated through graded alcohols to water. For antigen retrieval, sections were pre-treated in a pressure cooker, in 0.1 M citrate buffer (pH 6.0) for 1.5 min for Ki67, and 3 min for Mcm2 and Gem95. Endogenous peroxidase activity was inhibited using peroxidase-blocking solution (DakoCytomation, Glostrup, Denmark) for 10 min. Tris buffer saline (TBS) sections were incubated with primary antibody for 1 h at room temperature using the following dilutions: Mcm2 (1 : 1000), Ki67 (1 : 50) and Gem95 (1 : 800). The detection system was ChemMate Detection kit (DakoCytomation), which included application of biotinylated secondary antibody for 30 min and horseradish peroxidase for 30 min. Two washes with TBS were applied between the primary and secondary antibodies. Positive expression was visualised after 7 min incubation with DAB. Slides were washed in running tap water, counterstained with Mayer's haematoxylin and mounted with low-viscosity DPX mounting medium.

### Quantification method

Five representative fields per slide were examined at × 400 magnification under a light microscope with a rotating stage. Representative fields in OED were selected according to the most dysplastic areas and in OSCC were selected from the invasive front of the tumour. An eyepiece graticule was used and the epithelial–connective tissue interface was taken as the base of the square. All epithelial cells inside the area of the graticule were counted. Positive and negative epithelial cells and the total number of cells were recorded using a manual cell counter (The Denominator Co., CT, USA) and recorded in a computer. The labelling index (LI) was calculated by dividing the number of positive cells by the total number of cells per case and multiplying by 100. Intra-observer error was tested by re-examining 10% of the total number of cases. There were high levels of agreement according to the inter-class correlation coefficient for continuous data (*P*=0.00).

### Statistical analysis

Statistical analyses were carried out using the SPSS 14.0 and GraphPad InStat statistical software. Mcm2, Ki67 and geminin were normally distributed according to the Kolmogorov–Smirnov test. Although Mcm2/Ki67 and geminin/Ki67 ratios were normally distributed, because of their intrinsic constrained range of results these were treated as non-parametric. Therefore ANOVA and *t*-test analysis were used to evaluate differences between Mcm2, Ki67 and geminin LIs in the NOM, OED and OSCC groups. Mann–Whitney and Kruskal–Wallis tests were used to evaluate differences in the Mcm2/KI67 and geminin/Ki67 ratios. The general linear model (GLM) of repeated-measures analyses was used to evaluate the differences among the LI of the proteins (Mcm2, Ki67 and geminin) in every group (e.g. NOM or OED or OSCC). Scatter plots were used to observe the associations between proteins. Spearman's correlation coefficient was used to evaluate correlation between protein expression and grade of dysplasia.

## Results

In the group of OED that progressed to carcinoma the mean age of the patients was 59.9 (standard deviation (s.d.) ±15.6) with 23 men (56%) and 18 women (44%). Malignant transformation occurred within 6–200 months (mean 56.6±52.6). Twenty-nine lesions were graded as severe epithelial dysplasia, eleven as moderate and one as mild. In the group of OED with no history of malignant progression the mean age was 60.3 (±14.8) and there was a follow-up time of 54–349 months (mean 176.0±64); 25 (62.5%) were men and 15 (37.5%) were women. Twenty-three lesions were graded as severe epithelial dysplasia and 17 as moderate. Of the total 81 OED samples 1 was mild dysplasia, 28 were moderate dysplasias and 52 were severe dysplasias. The mean age of the OSCC group was 65.4 (±15.9). Five cases were early invasive OSCC, 22 were well differentiated, 10 were moderately differentiated and 1 was poorly differentiated. The mean age of the NOM cases was 37.2 (±19.1).

From the 134 cases, high-quality immunohistochemical staining was available in 131 samples for Mcm2, 129 samples for Ki67 and 133 samples for geminin.

### Expression of cell-cycle proteins in normal oral mucosa

Normal oral mucosa showed the lowest LI for Mcm2, Ki67 and geminin and a low geminin/Ki67 ratio when compared to the OED groups and OSCC. The Mcm2/Ki67 ratio showed that Mcm2 expression was higher than Ki67 with a ratio of 1.75 (Table 1).

**Figure 1:**
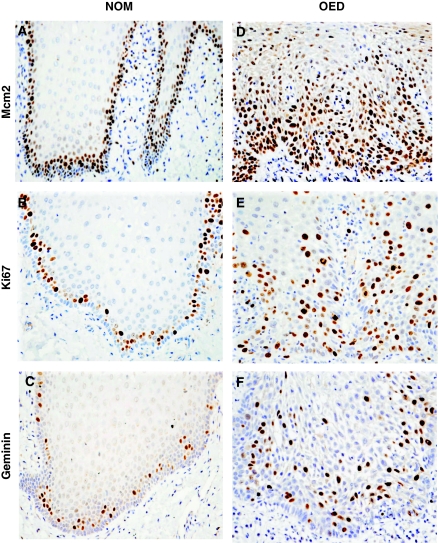
Immunoexpression pattern of proteins Mcm2 (**A**), Ki67 (**B**) and geminin (**C**) in NOM. Immunoexpression pattern of proteins Mcm2 (**D**), Ki67 (**E**) and geminin (**F**) in OED that progressed to OSCC. Original magnification × 400.

Mcm2, Ki67 and geminin protein were located mainly in the suprabasal compartment. In most of the NOM samples there was a characteristic absence of protein expression in the basal layer, specially for Ki67 and geminin (Figure 1A–C).

### Expression of cell-cycle proteins in oral epithelial dysplasias

In all OED samples there was a higher expression of Mcm2 when compared to Ki67 and geminin (Table 1; Figure 1D–F). Mcm2 expression extended from the basal and suprabasal compartments to the mid-prickle cell region (Figure 1D) and in some cases to the surface layers.

Expression of Mcm2 was higher (*P*=0.04) in the OED that progressed to OSCC than in those that did not progress (Table 2). In contrast, there was no significant difference in the geminin and Ki67 LIs between the two OED groups (Table 2). The localisation of Ki67 and geminin was expressed in the same areas as Mcm2, but in a significantly (GLM *P*<0.001) lower proportion of cells (Table 2; Figure 1E and F).

The Mcm2/Ki67 and geminin/Ki67 ratios (Table 2) were higher in the OED with malignant progression compared to the OED with no history of malignant transformation (*P*=0.04 and 0.02 respectively).

There was no correlation between the grade of dysplasia and the LI of the different proteins.

### Expression of cell-cycle proteins in oral squamous-cell carcinoma

**Figure 2:**
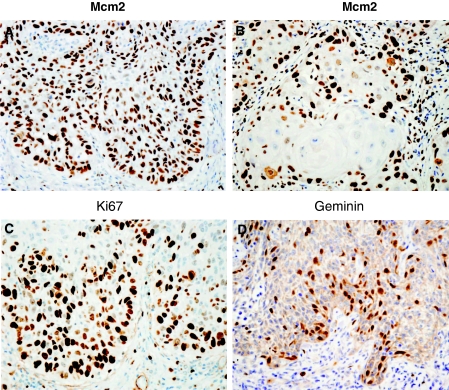
Invasive front of OSCC, immunoexpression pattern of proteins Mcm2 (**A**), Mcm2 around a keratin pearl (**B**), Ki67 (**C**) and geminin (**D**). Original magnification × 400.

Expression of Mcm2, Ki67 and geminin in the OSCC samples was seen in a high number of epithelial cells with stronger staining intensities at the invasive front (Figure 2A, C and D). When keratin pearls were present expression of Mcm2, Ki67 and geminin was seen around the periphery of the islands (Figure 2B).

The Mcm2/Ki67 ratio in OSCC was lower than in OED and NOM, but the geminin/Ki67 ratio was increased (Table 1).

There was no correlation between the level of tumour differentiation and the LI of the different proteins.

**Figure 3:**
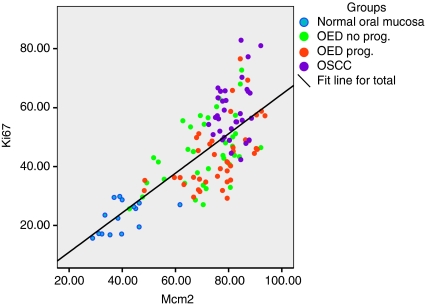
Scatter plot showing the positive association between the labelling indices for Mcm2 and Ki67 in NOM, OEDs and OSCC.

**Figure 4:**
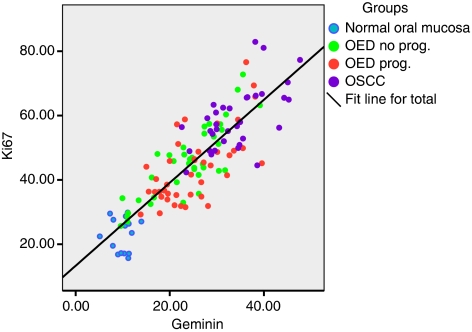
Scatter plot displaying the positive association between Ki67 and geminin labelling indices in NOM, OEDs and OSCC.

The LIs of Mcm2, Ki67 and geminin were progressively higher from NOM through OED to OSCC (Table 1; ANOVAs, *P*<0.001). In addition, there was a positive linear association between Mcm2 and Ki67 in all the analysed samples (Figure 3) as well as between geminin and Ki67 (Figure 4).

## Discussion

Others have described the importance of pre-replication proteins, especially Mcm2, as prognostic markers in dysplasias and neoplasia at various sites in the human body (Williams *et al*, 1998, 2004; Stoeber *et al*, 1999; Meng *et al*, 2001; Wharton *et al*, 2001; Alison *et al*, 2002; Going *et al*, 2002; Eward *et al*, 2004; Gonzalez *et al*, 2004; Dudderidge *et al*, 2005; Quaglia *et al*, 2006). In the oral mucosa only a few studies have explored this (Kodani *et al*, 2001, 2003; Scott *et al*, 2006). To the best of our knowledge, this is the first study in which geminin or Mcm2/Ki67 and geminin/Ki67 ratios have been evaluated in NOM or in oral dysplasias and carcinomas. Overall geminin and Mcm2 showed a clear association with proliferation (Ki67) and grade, the LI increased from normal mucosa to OED to OSCC (Figures 3 and 4).

The results of this study suggest that in NOM, most cells are in the G_0_ phase (not in cycle) with a lesser number in a licensed G_0_–G_1_ transition (demonstrated by Mcm2 expression). Only about 20% of cells are actually in the cell cycle (demonstrated by Ki67 and geminin expression). This suggests that the epithelial basal and suprabasal compartments in NOM have a low and controlled proliferation rate but with a continuous proliferative capacity. Low LIs in normal tissues have also been observed by other authors for Mcm2 and Ki67 in oral mucosa (Kodani *et al*, 2001), larynx (Chatrath *et al*, 2006) and prostate tissues (Meng *et al*, 2001), and for geminin in breast tissue (Shetty *et al*, 2005).

The absence of Mcm2 expression in a significant proportion of basal cells, and complete absence of Ki67 and geminin in this cell layer of NOM observed in this study, suggests that these cells are in a temporary G_0_ state. An *in vitro* study, using a fibroblast model system coupled to a cell-free DNA replication assay, identified a group of stem cells in intestinal epithelium that are unlicensed (Mcm2 negative) and with no geminin expression. According to the authors, these could be stem cells that may pass through a prolonged cell cycle as a defensive mechanism against possible genotoxic insult in rapidly proliferating tissues (Kingsbury *et al*, 2005). Similarly, a recent study (Takeda *et al*, 2006) has suggested that stem cells are present in the basal layer of NOM, based on expression of stem cell markers, cytokeratin 19 and p63. The low expression of Mcm2 in the basal layer of the normal oral epithelium was an unexpected finding but is in keeping with the suggestion of a self-defence mechanism to maintain a controlled cell proliferation of the oral mucosa.

In contrast to NOM, the levels of expression of Mcm2, geminin and Ki67 were elevated in OED indicating a constant cell-cycle re-entry with a high proportion of licensed and dividing cells. This has also been observed in studies of Mcm2 and Ki67 in Barrett's oesophagus (Going *et al*, 2002) and in neoplastic oesophagus using Mcm5 (Wharton *et al*, 2001).

The present study showed similar results to that of Kodani *et al* (2001), although the LIs for Mcm2 and Ki67 were higher in our study. The reason for this might be that Kodani *et al* quantified cells throughout the whole thickness of the epithelium, from basal to surface epithelial layers. Thus, the additional numbers of differentiated and non-expressing epithelial cells included in the upper third of the epithelium would have resulted in a lower overall percentage of positive expression and a lower LI. Quantification in the present study focused on the proliferative epithelial compartments where Mcm2, Ki67 and geminin are expressed more frequently. Other authors have evaluated Mcm2 and Ki67 in dysplastic lesions by dividing the epithelium into compartments. Nevertheless, the results from Kodani *et al* were similar to those reported here and showed a comparable increase in expression from NOM to OSCC.

Although these results need to be confirmed by other similar studies, Ki67 and geminin showed no significant difference between the OED groups, however Mcm2 had a marginally but significantly higher mean LI, in the OED that progressed to OSCC than the OED with no history of malignant progression, this was also observed previously (Kodani *et al*, 2001). These findings could suggest that cells in OED with malignant progression have an increased number of cells licensed to proliferate. Ibarra *et al* (2008) observed that a full complement of MCM proteins is essential to protect cells from the natural replicative stresses during S phase to maintain genome integrity. Therefore, the high levels of Mcm2 in the OEDs could suggest a similar defensive mechanism during genomic damage before malignant transformation occurs.

The Mcm2/Ki67 ratio was higher in OED that progressed to OSCC compared to OED with no malignant progression due to the high levels of Mcm2 in the first group. Likewise, the geminin/Ki67 ratio was higher, indicating a shorter G_1_ phase and an increased proliferation rate. Although the Mcm2/Ki67 and geminin/Ki67 ratios were significantly different between the OED with and without malignant progression (Table 2), the differences in the values are relatively small and this finding needs to be confirmed in further similar studies.

The results suggest that cells in oral squamous-cell carcinoma are in a high proliferation state as demonstrated by the high levels of Mcm2, geminin and Ki67 (Table 1; Figure 2). Although the LI for Mcm2 in the present study indicates that it is an effective proliferation marker, Ki67 showed the biggest difference in values from an LI of 44.55 in OED to 59.03 in OSCC (Table 1). A significant rise in Ki67 expression has also been observed in regenerative nodular lesions of cirrhotic liver that progressed to hepatocellular carcinoma, but not for Mcm2 and geminin (Quaglia *et al*, 2006).

The invasive front of OSCC was selected for protein evaluation, because this is considered to be more relevant for the understanding of the cell-cycle kinetics of cells involved with invasion into the adjacent tissues. This method may also be more reproducible as it is easier to standardise evaluation of the inferior limit (invasive front) of the lesion between different observers.

This is the first time that elevated geminin expression has been reported in OSCC (Table 1). This finding is consistent with previous immunohistochemical, immunoblotting and *in situ* hybridisation studies where geminin is overexpressed in cancer cell lines (Xouri *et al*, 2004) and unveiled as a proliferation marker in different human cancers (Wohlschlegel *et al*, 2002).

Further studies of OSCC evaluating Mcm2, Ki67 and geminin expression, along with their Mcm2/Ki67 and geminin/Ki67 LI ratios, may provide further evidence of their prognostic potential, especially if they can be correlated with mode of invasion, tumour size and cumulative disease-free survival.

## Conclusions

Overall the LI of all proteins increased progressively from NOM to OED to OSCC. Geminin has been shown as a proliferative marker in these oral tissues. Elevated levels of expression of Mcm2, Ki67 and geminin in OED suggest a constant cell-cycle re-entry. We also observed that Mcm2 as a proliferation marker may be superior to Ki67 because it indicates licensed capacity. In addition, Mcm2 was marginally but significantly more elevated in OED that progressed to OSCC. The findings reported here require confirmation from further studies; however, information on Mcm2, Ki67 and geminin may be of prognostic value in OEDs.

## Figures and Tables

**Table 1:** Mean and s.d. of Mcm2, Ki67 and geminin LI and median with percentiles in the Mcm2/Ki67 and geminin/Ki67 ratios in NOM, all OEDs and OSCC

**Labelling index mean (s.d.)**					**Median (25–75%)**	
	**Mcm2**	**Ki67**	**Geminin**	**Stat. GLM**	**Mcm2/Ki67 ratio**	**Geminin/Ki67 ratio**
NOM (*n*=15)	39.94 (8.1)	22.94 (5.2)	9.95 (2.1)	*P*<0.001	1.75 (1.42–2.11)	0.43 (0.37–0.56)
OED (*n*=81)	73.62 (12.1)	44.55 (11.1)	24.40 (7.2)	*P*<0.001	1.75 (1.42–1.97)^a^	0.54 (0.47–0.62)
OSCC (*n*=38)	81.16 (4.9)	59.03 (9.6)	34.08 (6.3)	*P*<0.001	1.36 (1.23–1.55)	0.58 (0.51–0.62)
ANOVA test	*P*<0.001	*P*<0.001	*P*<0.001		*P*⧫=0.00	*P*⧫=0.01

Abbreviations: All OED=all oral epithelial dysplasias (OED that progressed and did not progress to OSCC); GLM=general linear model; NOM=normal oral mucosa; OSCC=oral squamous-cell carcinomas from progressing OED; *P*⧫=Kruskal-Wallis test; ANOVA=analysis of variance.

a *Note*: The median Mcm2/Ki67 OED has a lower 75 percentile than NOM, therefore it has proportionally fewer high values, meaning it is actually lower hence the Kruskal–Wallis test.

**Table 2:** Mean and s.d. of Mcm2, Ki67 and geminin LIs and median with percentiles in the Mcm2/Ki67 and geminin/Ki67 ratios in OEDs that progressed to carcinoma and in OEDs with no malignant progression

**Labelling index mean (s.d.)**					**Median (25–75%)**	
	**Mcm2**	**Ki67**	**Geminin**	**Stat. GLM**	**Mcm2/Ki67 ratio**	**Geminin/Ki67 ratio**
OED no progr. (*n*=40)	70.74 (12.84)	45.23 (11.17)	23.71 (7.0)	*P*<0.001	1.63 (1.28–1.86)	0.51 (0.44–0.58)
OED progr. (*n*=41)	76.28 (10.90)	43.90 (11.17)	25.06 (7.5)	*P*<0.001	1.81 (1.52–2.03)	0.58 (0.48–0.68)
*t*-test	*P*=0.04	NS	NS		*P*▪=0.04	*P*▪=0.02

Abbreviations: GLM=general linear model; OED no prog.=oral epithelial dysplasias with no history of malignant progression; OED prog.=oral epithelial dysplasias that progressed to oral squamous-cell carcinoma; NS=not significant; *P*▪=Mann–Whitney test.
